# Advanced approaches of the use of circRNAs as a replacement for cancer therapy

**DOI:** 10.1016/j.ncrna.2024.03.012

**Published:** 2024-03-30

**Authors:** Goran Sedeeq Hama Faraj, Bashdar Mahmud Hussen, Snur Rasool Abdullah, Mohammed Fatih Rasul, Yasaman Hajiesmaeili, Aria Baniahmad, Mohammad Taheri

**Affiliations:** a Department of Medical Laboratory Science, Komar University of Science and Technology, Sulaymaniyah, 46001, Iraq; b Department of Biomedical Sciences, College of Science, Cihan University-Erbil, Erbil, Kurdistan Region, 44001, Iraq; c Department of Clinical Analysis, College of Pharmacy, Hawler Medical University, Erbil, Kurdistan Region, 44001, Iraq; d Medical Laboratory Science, Lebanese French University, Erbil, Kurdistan Region, 44001, Iraq; e Department of Pharmaceutical Basic Science, Faculty of Pharmacy, Tishk International University, Erbil, Kurdistan Region, Iraq; f Faculty of Health, York University, Toronto, ON, Canada; g Institute of Human Genetics, Jena University Hospital, Jena, Germany; h Urology and Nephrology Research Center, Shahid Beheshti University of Medical Sciences, Tehran, Iran

**Keywords:** Cancer, Cancer therapy, CircRNAs, Challenges, Strategies

## Abstract

Cancer is a broad name for a group of diseases in which abnormal cells grow out of control and are characterized by their complexity and recurrence. Although there has been progress in cancer therapy with the entry of precision medicine and immunotherapy, cancer incidence rates have increased globally. Non-coding RNAs in the form of circular RNAs (circRNAs) play crucial roles in the pathogenesis, clinical diagnosis, and therapy of different diseases, including cancer. According to recent studies, circRNAs appear to serve as accurate indicators and therapeutic targets for cancer treatment. However, circRNAs are promising candidates for cutting-edge cancer therapy because of their distinctive circular structure, stability, and wide range of capabilities; many challenges persist that decrease the applications of circRNA-based cancer therapeutics. Here, we explore the roles of circRNAs as a replacement for cancer therapy, highlight the main challenges facing circRNA-based cancer therapies, and discuss the key strategies to overcome these challenges to improve advanced innovative therapies based on circRNAs with long-term health effects.

## 1 Introduction

Cancer continues to be one of the most challenging problems in modern medicine due to its complex molecular pathways and the ongoing evolution of therapeutic resistance [1]. There have been considerable advancements in the treatment of different cancer types using conventional cancer therapies like chemotherapy and radiation [2]. However, these methods frequently have serious side effects and have restrictions on the molecular triggers of cancer growth that they can target [3]. In recent years, circRNAs have attracted much attention as a fresh and promising approach to cancer treatment.

CircRNAs are novel-type non-coding RNA molecules that are covalently closed, single-stranded RNA molecules without 5′–3′ end and poly (A) tails. They are more stable as they resist exonuclease-mediated destruction compared to linear transcripts [4]. Structurally, circRNAs consist of exons or introns, and circRNAs with exons are frequently found in the cytosol, while circRNAs with introns are primarily found in the nucleus [5]. Linking the 3′ downstream terminal with the 5′ upstream terminal through a back-splicing process creates its circular structure [6]. This circular structure of circRNA helps it to be protected from destruction by exonucleases and makes circRNAs more stable. Therefore, the use of circRNA in the field of disease therapy, especially cancer therapy, is progressing in the right direction [7].

Numerous circRNAs have been found due to the advancement of bioinformatics techniques and high-throughput RNA sequencing (RNA-seq). For instance, in human fibroblasts, Jeck et al. observed over 25,000 circRNAs that have not been broken down by exonucleases [8]. Similarly, in samples of juvenile acute lymphoblastic leukemia, spliced gene transcripts make up a large percentage of the circRNA map, as Salzman and his colleagues explored [9].

Synthetic circRNAs are being investigated for application in disease therapy, and recently, there has been an increase in interest in developing technology for their synthesis [10]. Synthetic circRNAs have also been used as biosensors and therapies, such as the replacement of therapeutic proteins and peptides [11] and vaccinations [12].

Despite their unique properties as potential cancer treatments, such as their circular structure and stability, several obstacles still stand in the way of their extensive therapeutic applications. This review highlights the functions of circRNAs as an alternative for cancer therapy, identifies the most critical challenges plaguing circRNA-based cancer therapies, and addresses the essential techniques to overcome these challenges to enhance advanced innovative therapeutics based on circRNAs with long-term health impacts.

## 2 CircRNA biogenesis and characterization

CircRNAs are created through non-canonical splicing processes called back-splicing, which is classified as an alternative splicing process. Cellular spliceosomal machinery is necessary to synthesize circRNAs in humans and animals [13]. A back-splicing process joins an upstream 3′ splice site with a downstream 5′ splice site to make a single-strand, covalently closed-loop structure [14]. This is how most circRNAs are made from pre-mRNAs. Different circRNAs can be made from identical sequences using alternate back-splicing [10].

Despite extensive study over many years, the specific mechanism underlying circRNA synthesis remains unknown. According to their cycling mechanisms and composition, circRNAs are typically categorized into three kinds: exonic circRNAs (ecirRNAs) [15], intronic circRNAs (ciRNAs) [16], and exon-intron circRNAs (EIciRNAs) [17]. Exonic circRNAs are primarily found in the cytoplasm and have one or more exons, with two or three exons coming from alternative splicing [18]. There are many types of nuclear back-splicing and linear splicing processes that have been studied. Some of these are exon skipping, intron pairing, and RNA-binding proteins (RBPs) [19]. Recently, it has been discovered that a novel class of mitochondrial-encoded circRNAs (mecciRNAs) acts as molecular chaperones to assist the mitochondrial entrance of nuclear-encoded proteins [20].

**Fig. 1:**
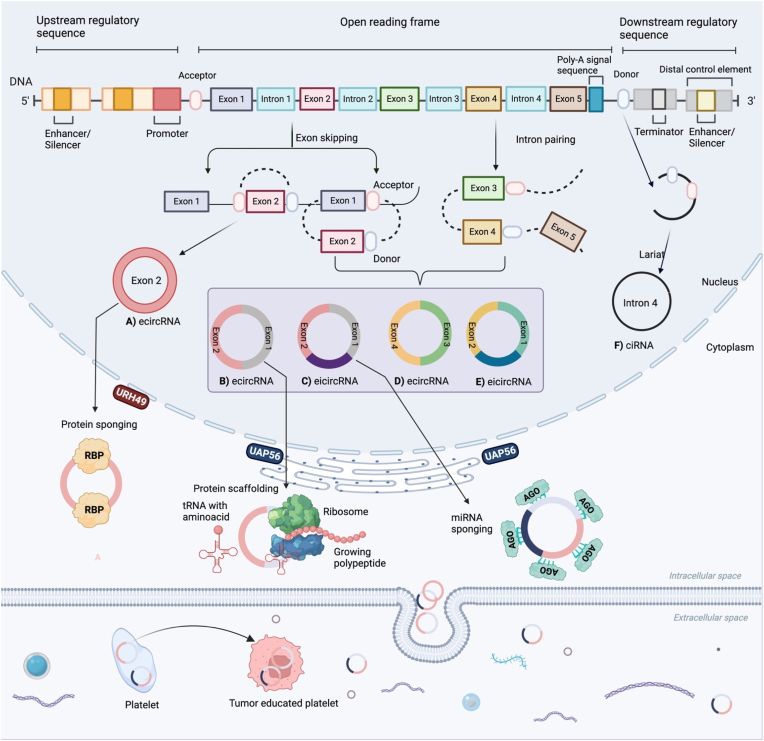
Formation of circRNA and the three main hypotheses: an ecircRNA is produced when introns are removed through intron-pairing-driven circularization, also called direct back-splicing. In this process, the pre-flanking mRNA's intronic complementary sequences create a lariat through direct base pairing. A process of circularization known as exon-skipping or lariat-driven produces pre-ecircRNA by removing transcript introns. RBP-mediated circularization regulates circRNA production by managing activators and inhibitors. *RBP* RNA binding proteins, *URH49* uridine-ribohydrolase 49, *tRNA* transfer RNA, *AGO* Argonaute protein.

Three possible mechanisms for circRNA formation have been proposed: First, ecirRNAs are primarily formed through direct back-splicing, also known as intron-pairing-driven circularization, in which the pre-flanking mRNA's intronic complementary sequences form a lariat by directly base-pairing, which results in an ecircRNA upon intron removal [17]. Secondly, RNAs fold close to exons and connect the downstream splicing donor to the upstream splicing acceptor through 3′, 5′ phosphodiester bonds to create a lariat structure, secondary splicing, or intron splicing to make a loop, and then cut introns to make an exon-containing lariat. This is also known as exon-skipping. Furthermore, intron lariats with a branching point rich in cytosine and a 5′ splice site rich in 7-nucleotide guanine uracil can produce ciRNAs without being impacted by debranching enzymes [21]. Lastly, in RBP-mediated circularization, the production of circRNA is significantly regulated by RBPs that act as managing activators or inhibitors (Fig. 1). RNA editing enzyme ADR1 stops the output of circRNA by directly inverting ALU repeats. This is mediated by A-to-I editing of RNA pairing circularized exons [22,23]. This reduces the complementarity and stability of intron-base pairing interactions [18]. Currently, numerous studies have revealed the aberrant circRNA expression patterns and their regulatory roles in the development and spread of cancer [24,25].

## 3 Innovative advances and therapeutic potential of circRNA-based cancer therapy

Single-stranded and covalently closed circRNA molecules were initially described in the genomes of virions by Sanger et al., in 1976 [26]. Later, in 1979, Hsu et al. proved circRNAs without free terminals and the need for companion protein [27]. In 1991, Nigro et al. became the first to describe how non-canonical splicing led to the unexpected isolation of isoforms from the deleted colon cancer gene (“scrambled exons”) [28]. Further, Cocquerelle and his team presented consistent findings for the human EST-1 gene in 1992, and they established a link between the presence of many nearby introns and the synthesis of these transcripts [29]. They showed that the circularized RNAs remained stable in the cytoplasm over two days after actinomycin D treatment [30]. Later studies by Cape et al. found that the scrambled product of gene Sry RNA was circular; this circRNA is primarily intracellular, tissue-specific, and present in three different mouse strains [31]. When these hypothetical steps have been completed, nuclear extracts have been shown in several studies to be useful for the in vitro generation of circRNAs, the target molecules for this chemical [32,33].

**Fig. 2:**
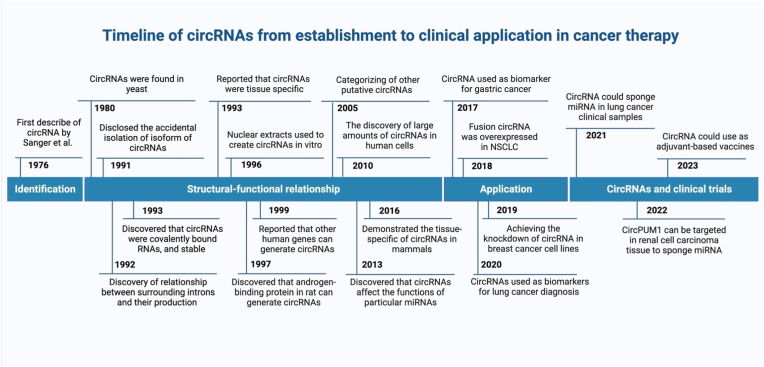
A timeline outlining the most significant findings made about circRNA-based cancer therapy.

From 1996 to the beginning of the 2000s, scientists discovered that other human genes could make circRNAs. For example, cytochrome P450 2C24 in rats [34], cytochrome P450 in humans, androgen-binding protein in the rat [35], dystrophin in humans [36], and cyclin-dependent kinase 4 (INK4/ARF)-associated ncRNA were used to make other types of circRNA [37]. Even though these early studies proved that RNA molecules can fold back on themselves, their significance was not immediately recognized. Advances in RNA-seq technologies and specialized computational workflows prompted a new wave of circRNA research in 2010. Later, circRNAs were found to affect the actions of particular miRNAs and were subsequently used as a biomarker in diagnosing cancer such as lung cancer [38]. Recently, circRNAs were studied in clinical trials, such as circPUM1 targets in renal cell carcinoma tissue to sponge miRNA [39] (Fig. 2). These circular RNA molecules have proven to be capable of controlling gene expression, interacting with different cellular elements, and acting as valuable biomarkers. As this research field develops, circRNA-based therapeutics have the potential to offer cancer patients more effective and individualized therapy alternatives.

## 4 CircRNAs' potential roles in carcinogenesis

CircRNAs, a family of long non-coding RNAs, are involved in several critical biological processes that promote or inhibit cancer [[40], [41], [42]]. More evidence suggests that circRNAs play a crucial role through different mechanisms in several malignancies, including esophagus cancer (EC) [43], lung cancer (LC) [44], gastric cancer (GC) [45], breast cancer (BC) [46], and colorectal cancer (CRC) [47,48].

The physiological functions of circRNAs are mediated by the miRNA sponge in cancers. For instance, circBCAR3, a molecular sponge for miR-27a-3p, increases tumorigenesis and metastasis in EC patients [43]. Likewise, hsa-circ-0013958 has been upregulated in plasma and tissues of LC patients, and sponging miR-134 leads to elevated levels of cyclin D1, a known carcinogenic protein. Further, the hsa-circ-0013958 expression level was connected with lymphatic metastasis and TNM stage [49]. Moreover, overexpression of hsa_circRNA_102958 increases the proliferation of GC, although its expression level was associated with the TNM stage [50]. Similarly, hypoxia-inducible circWSB1 was significantly upregulated and interacts with USP10 to reduce the stability of p53 caused by USP1 and promote the progression of BC tissues [46]. Furthermore, circTDRD3 was upregulated under hypoxic conditions and accelerates the progression and spread of CRC by affecting a positive feedback loop through the HIF1α/PTBP1/circTDRD3/miR-1231/HIF1α pathway [47]. The molecular mechanisms that lead to tumors progressing to a malignant state may be better understood in light of these results.

Consequently, the abundance of stable circRNAs constitutes a new class of RNA species that may distinguish between cancer cells and healthy cells, proving its significant potential as a circulating biomarker for diagnostic cancers. The exact role of circRNAs in carcinogenesis remains unknown despite recent progress in this field.

According to growing data, circRNAs may be employed as prospective genetic markers for diagnostic techniques, prognosis, early cancer recognition, and even therapy response monitoring. The following are the principal applications of circRNAs in cancer.

### 4.1 CircRNAs as a biomarker in cancer diagnosis and therapy

**Fig. 3:**
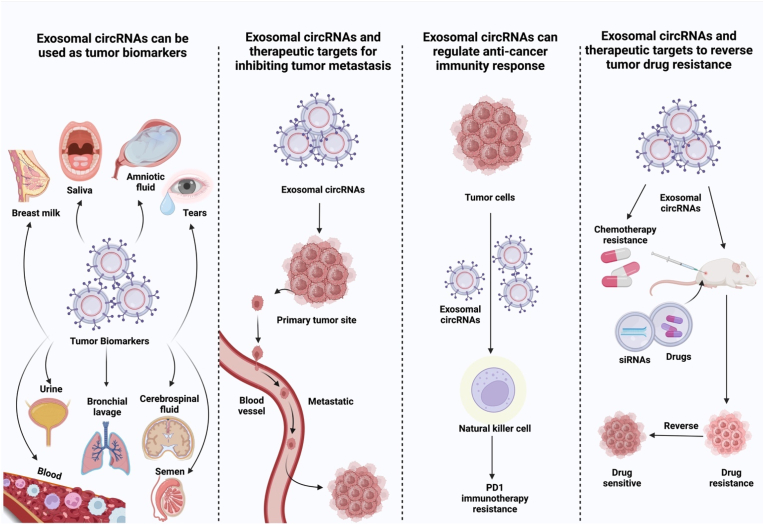
Use of circular RNAs (circRNAs) as diagnostic and prognostic biomarkers and as possible therapeutic interventions for human diseases and disorders, including cancer, biological samples that contain circRNAs are tears, saliva, cerebrospinal fluid, bronchial lavage, breast milk, blood, amniotic fluid, urine, and semen.

CircRNAs can serve as cancer biomarkers due to their dysregulation and association with cancer morphologies. CircRNAs have a high degree of tissue- and disease-specificity, making them a potential choice for cancer diagnostics [51] (Fig. 3). More evidence suggests that circRNAs are involved as a biomarker in diagnosing several cancers. For instance, cir-ITCH acts as a sponge for miR-7/17/214 in ESCC, which may increase the level of ITCH circRNA. ITCH overexpression promotes the ubiquitination and degradation of phosphorylated Dvl2, which in turn limits the progression of ESCC by inhibiting the Wnt/β-catenin axis [52]. Likewise, hsa_circ_0013958 up-regulates oncogenic cyclin D1 through sponging of miR-134, which plays an essential role in the progression of NSCLC. According to these findings, hsa circ 0013958 might be employed as a non-invasive biomarker for early diagnosing and screening LAC [49]. Moreover, Li et al. revealed that the sensitivity and specificity of identifying hsa circ 0001649 between GC and normal samples are satisfactory. This means that it could be used as a biomarker for non-invasive screening of GC by making comparisons of the expression profiles in tissue and serum samples [53].

CircRNAs can act as a competitive endogenous RNA (ceRNA) to inhibit specific target genes in several types of cancer, which might be used as a diagnostic biomarker. For example, circGFRA1 acts as a ceRNA to control *GFRA1* production by sponging miR-34a in TNBC and could be used as a diagnostic biomarker and a potential target for TNBC treatment [54]. Similarly, circ-PDE8A regulates MACC1 and promotes invasive development via the MACC/MET/ERK or AKT pathways in PDAC by acting as a ceRNA for miR-338. It is suggested that exosomal circ-PDE8A could be a valuable diagnostic for PDAC diagnosis or prognosis, and circ-PDE8A plays a crucial role in tumor invasion [55]. These data suggest that tumor cell-derived circRNAs are released into the tumor milieu surrounding the circulation, supporting the use of circRNAs as biomarkers for patients with malignant tumors [56].

According to recent studies, circRNAs can be utilized to predict the metastasis of cancer cells. For instance, in a study comparing the circRNA regulation profiles of six patients, Xu et al. showed that circRNA 0001178 and circRNA 0000826 were considerably overexpressed in CRC metastatic tissue samples, allowing them to reliably identify between CRC and liver metastasis patients [57]. Moreover, circASAP1 was markedly upregulated in the HCC tissues of patients with lung metastasis after surgery. Downward regulation of circASAP1 was associated with decreased overall survival and an increased recurrence rate and was positively correlated with CSF1, MAPK1, and CD68^+^ tumor-associated macrophage levels [58]. Thus, circASAP1 could have a potential role in the prognosis of HCC metastasis.

Based on other studies, circRNAs are essential for predicting therapeutic resistance in a specific type of cancer. For instance, cisplatin is one of the most effective chemotherapeutics for treating GC [59]. However, Huang et al. showed that circAKT3 upregulates *PIK3R1*, which promotes cisplatin resistance via sponging of miR-198, in 105 GC patients [60]. This clarified that circAKT3 is a highly reliable prognostic biomarker in GC patients, which makes them resistant to cisplatin. Similarly, circ_0076305 has been proven to increase *ABCC1* expression by sponging miR-186-5p, driving resistance to cisplatin in NSCLC [61]. CircRNAs are more specifically described in Table 1 as prognostic and diagnostic biomarkers in several cancer types.

**Table 1:** CircRNAs as potential indicators for cancer diagnosis and prognosis.

Type of cancer	circRNA	Source	Biomarker	Regulation	miRNA sponge	Mechanism	Refs
LC	hsa-circ-0013958	Tissue, plasma, cell line	Diagnostic in NSCLC	↑	miR-134	hsa-circ-0013958↑- miR-134↓-cyclin-D1↑	[49]
LC	hsa_circ_0075930	Cell line, tissue	Diagnostic in NSCLC	↑	miR-149-5p	–	[62]
LC	ciRS-7	Tissue	Prognostic biomarker in NSCLC	↑	miR-7	–	[63]
LC	circFARSA	Tissue, plasma	Diagnostic in NSCLC	↑	miR-330-5p and miR-326		[64]
LC	circPRMT5	Tissue, cell line	Diagnostic in NSCLC	↑	miR-377, miR-382, and miR-498	–	[65]
LC	circRNA100146	Tissue	Diagnostic	↑	miR-361-3p and miR-615-5p	circRNA100146↑- miR361-3p ↓/miR-615-5p↓- SF3B3↑	[66]
LC	circ_0005280	Tissue	Diagnostic	↓	–	Unknown	[67]
LC	circRNA_102231	Tissue	Diagnostic	↑	Unknown	Unknown	[68]
LC	circ-ITCH	Tissue	Diagnostic	↓	miR-7 and miR-214	circ-ITCH↓- miR-7↑/miR214↑-Wnt/β-catenin↑	[69]
LC	circPVT1	NSCLC tissue, cell line	Diagnostic	↑	miR-125b	circPVT1↑-miR-125b↓- E2F2 pathway↑	[70]
LC	circMET	MSCLC tissue, cell line	Diagnostic	↑	miR-145-5p	circMET↑-miR-145-5p↓- CXCL3↑	[71]
LC	circGFRA1	MSCLC tissue, cell line	Diagnostic	↑	miR-188-3p	circGFRA1↑-miR-188-3p↓- PI3K/AKT↑	[72]
LC	hsa_circ_0001946	Tissue, cell line	Diagnostic	↓	miR-135a-5p	hsa_circ_0001946↓-NER signaling pathway↑	[73,74]
LC	hsa_circ_0030998	Tissue	Diagnostic	↓	miR −558	hsa_circ_0030998↓-miR −558↑-MMP1/MMP17↓	[75]
LC	circ-CCS	Tissue	Diagnostic	↑	miR-383	circ-CCS↑-miR-383↓- E2F7↑	[76]
LC	circ-IGF1R	Tissue, cell line	Diagnostic	↓	miR-1270	circ-IGF1R↓–miR-1270↑– VANGL2↓	[77]
LC	circRNA_102179	Tissue, cell line	Diagnostic	↑	miR-330-5p	circRNA_102179↑-miR -330-5p↓-HMGB3↑	[78]
LC	circ-ZKSCAN1	NSCLC sample, cell line	Diagnostic	↑	miR-330-5p	circ-ZKSCAN1↑-miR-330- 5p↓-FAM83A↑	[79]
LC	hsa_circ_0007059	Tissue	Diagnostic	↓	miR −378	hsa_circ_0007059↓-miR −378↑-Wnt/β-catenin↑/ERK1/2↑	[80]
LC	circ-PITX1	Tissue	Diagnostic	↑	miR-1248	circ-PITX1↑-miR-1248↓- CCND2↑	[81]
LC	circ_0000429	NSCLC tissue, cell line	Diagnostic	↑	miR-1197	circ_0000429↑-miR-1197↓- MADD↑	[82]
LC	circ_0001287	Tissue, cell line	Diagnostic	↓	miR-21	circ_0001287↓-miR-21↑- PTEN↓	[83]
LC	hsa_circ_0000064	Tissue, cell line	Diagnostic	↓	–	hsa_circ_0000064↑- caspase-3/9↑/bax↑/p21↑/CDK6↑/cyclin D1↑/bcl-2↓/MMP-2/9↑	[84]
LC	circFGFR3	NSCLC tissue	Prognostic	↑	miR-22-3p	circFGFR3↑- miR-22-3p↓-Gal-1↑/p-AKT↑/p-ERK1/2↑	[85]
LC	circ_0003645	NSCLC tissue	Prognostic	↑	miR −1179	circ_0003645↑-miR −1179↓-TMEM14A↑	[86]
LC	CDR1as	Tissue	Prognostic	↑	miR-7	CDR1as↑-miR-7↓- EGFR↑/CCNE1↑/PIK3CD↑	[87]
LC	circ_POLA2	Tissue	Prognostic	↑	miR −326	circ_POLA2↑-miR −326↓-GNB1↑	[88]
LC	circ-FOXM1	Tissue	Prognostic	↑	miR -1304-5p	circ-FOXM1↑-miR -1304-5p↓-PPDPF↑/MACC1↑	[89]
LC	circPIP5K1A	Tissue, serum	Prognostic	↑	miR-600 and miR-101	circPIP5K1A↑- miR-600↓–HIF–1α↑ circPIP5K1A↑- miR-101↓-ABCC1↑	[90,91]
LC	circRNA_010763	Tissue	Prognostic	↑	miR-715	circRNA_010763↑- miR-715↓-*c*-Myc↑	[92]
LC	circRNA_100876	Tissue	Prognostic	↑	Unknown	Unknown	[93]
LC	circ-ANXA7	Tissue	Prognostic	↑	miR-331	circ-ANXA7↑- miR-331↓-LAD1↑	[94]
LC	circ-PTEN	Tissue, serum	Prognostic	↓	miR −155 and miR-330-3p	circ-PTEN↓-miR −155↑/miR-330-3p↑- PTEN↓	[95]
LC	hsa_circ_0008003	Tissue	Prognostic	↑	miR-488	hsa_circ_0008003↑- miR-488↓-ZNF281↑	[96]
LC	circ-MTHFD2	Tissue	Prognostic	↑	Unknown	Unknown	[97]
GC	hsa_circ_0000745	Plasma	Diagnostic	↓	–	–	[98]
GC	hsa_circ_0000520	Tissue, plasma	Diagnostic	↓	–	–	[99]
GC	hsa_circ_0014717	Tissue	Diagnostic	↓	–	–	[100]
GC	hsa_circ_0000096	Tissue	Diagnostic	↓	–	–	[101]
GC	hsa_circ_0066444	Tissue	Diagnostic	↑	–	–	[102]
GC	hsa_circRNA_102958	Tissue	Diagnostic	↑	–	–	[50]
GC	circLARP4	Tissue	Prognostic	↓	miR-424-5p	circ-RNA-LARP4↓-miR-424-5p↑-LATS1↓	[103]
GC	hsa_circ_0001368	Tissue	Prognostic	↓	miR-6506-5p	hsa-circ-0001368↓-miR-6506↑-FOXO3↓	[104]
GC	circPVT1	Tissue	Prognostic	↑	–	–	[105]
GC	circNRIP1	Tissue	Prognostic	↑	miR-149-5p	circNRIP1↑- miR-149-5p↓-AKT1/mTOR↑	[106]
GC	circ-KIAA1244	Plasma	Prognosis	↓	–	–	[107]
GC	circAKT3	Tissue	Prognostic	↑	miR-198	circAKT3↑- miR-198↓-PIK3R1↑	[60]
GC	hsa_circ_0010882	Tissue	Prognostic	↑	–	hsa_circ_0010882↑- p-PI3K, *p*-Akt, and *p*-mTOR↑	[108]
GC	circ-ATAD1	Tissue	Prognostic	↑	miR-140-3p	circ-ATAD1↑-miR-140-3p↓-YY1↑	[109]
GC	circ-HIPK3	Tissue	Prognostic	↑	miR-124/29b	–	[110]
GC	circ-DCAF6	Tissue	Prognostic	↑	miR-1231/1256	–	[111]
GC	circPVT1	Tissue	Prognostic	↑	miR-125	–	[105]
GC	circ-OSBPL10	Tissue	Prognostic	↑	miR-136-5p	–	[112]
GC	circ-NHSL1	Tissue	Prognostic	↑	miR-1306-3p	–	[113]
GC	circ-NRIP1	Tissue	Prognostic	↑	miR-149-5p	circ-NRIP1↑-miR-149-5p↓- AKT1/mTOR↑	[106]
GC	circ-LARP4	Tissue	Prognostic	↓	miR-424-5p	circ-LARP4 ↓-miR-424-5p↑-LATS1↓	[103]
GC	circ-LMTK2	Tissue	Prognostic	↓	–	–	[114]
GC	circ-PVRL3	Tissue	Prognostic	↓	–	–	[115]
GC	circ-CCDC9	Tissue	Prognostic	↓	miR-6792-3p	circ-CCDC9↓- miR-6792-3p↑-CAV1↓	[116]
CRC	Circ-VAPA	Plasma	Diagnostic	↑	miR-101	circVAPA↑- miR-101↑-	[117]
CRC	circITGA7	Tissue	Diagnostic	↓	miR-370-3p	hsa_circ_0026782↓-miR-370-3p↑-NF1↓	[118]
CRC	circHIPK3	Tissue	Prognostic	↑	miR-7	circHIPK3↑-miR-7↓-FAK, IGF1R, EGFR, YY1↑	[119]
CRC	ciRS-7	Tissue	Prognostic	↑	miR-7	–	[120]
CRC	circRNA_100290	Tissue, cell line	Prognostic	↑	miR-5166	circRNA_100290↑-miR-5166↓-Wnt/β-catenin↑	[121]
CRC	CircCCDC66	Tissue, cell line	Diagnostic	↑	–	–	[122]
CRC	hsa-circ-0136666	Tissue, cell line	Prognostic and prognostic	↑	miR-136	–	[123]
CRC	hsa-circ-0001649	Tissue, serum	Diagnostic	↓	–	–	[124]
CRC	hsa-circ-0000711	Tissue, cell line	Diagnostic and prognostic	↓	–	–	[125]
CRC	hsa-circ-0014717	Tissue, cell line	Prognostic	↓	–	–	[126]
CRC	hsa-circ-0000567	Tissue, cell line	Diagnostic	↓	–	–	[127]
CRC	hsa-circ-001988	Tissue	Diagnostic	↓	–	–	[128]
CRC	hsa-circ-26344	Tissue	Prognostic	↓	miR-21, miR-31	–	[129]
CRC	hsa-circ-003906	Tissue, cell line	Diagnostic	↓	–	–	[130]
CRC	hsa-circ-104700	Tissue	Diagnostic	↓	–	–	[131]
BC	hsa_circRNA_002178	Tissue	Prognostic	↑	miR-328-3p	hsa_circRNA_002178↑- miR-328-3p↓-COL1A1↑	[132]
BC	circ_0005230	Tissue	Prognostic	↑	miR-618	circ_0005230↑-miR-618↓-CBX8↑	[133]
BC	circKIF4A	Tissue	Prognostic	↑	miR-375	circKIF4A↑-miR-375↓- KIF4A↑	[134]
BC	circGFRA1	Tissue	Prognostic	↑	miR-34a	circGFRA1↑-miR-34a↓- GFRA1↑	[54]
HCC	Circ-CDYL	Tissue	Diagnostic	↑	–	–	[135]
HCC	circSMARCA5	Plasma	Diagnostic	↓	–	–	[136]
HCC	hsa_circ_0000976, hsa_circ_0007750 and hsa_circ_0139897	Plasma	Diagnostic	↑	–	–	[137]
HCC	circ_104075	Tissue	Diagnostic	↓	miR-582-3p	circ_104075 ↑-miR-582-3p↓-YAP↑	[138]
HCC	circZKSCAN1	Tissue	Diagnostic	↓	–	–	[139]
HCC	circ-10720	Tissue	Prognostic	↑	–	–	[140]
HCC	circRNA 101368	Tissue	Prognostic	↑	miR-200a	circRNA 101368↑-miR-200a↓-HMGB1/RAGE↓	[141]
HCC	circRNA-100338	Tissue	Prognostic	↑	miR-141-3p	circRNA-100338↑-miR-141-3p↓ MTSS1↑	[142]
HCC	SCD-circRNA 2	Tissue	Prognostic	↑	–	–	[143]
HCC	circADAMTS13	Tissue	Prognostic	↓	miR-484	–	[144]
PC	circ-LDLRAD3	Plasma	Diagnostic	↑	–	–	[145]
PC	circ-PDE8A	Plasma	Prognostic	↑	miR-338	circ-PDE8A↑- miR-338↓-MET↑	[55]
PC	circ-IARS	Tissue	Prognostic	↑	miR-122	circ-IARS↑- miR-122↓- RhoA and RhoA-GTP↑	[146]
PCa	CircTENM3	Tissue	Diagnostic	↓	miR-558	CircTENM3↑- miR-558↓- RUNX3↑	[147]
PCa	circFGFR^1int2^	Cell line/Tissue	Diagnostic	↑	miR-4687-5p	circFGFR^1int2^↑- miR-4687-5p↓-P65/FUS↑	[148]
PCa	circROBO1	Cell line/Tissue	Diagnostic	↑	miR-556-5p	circROBO1↑- miR-556-5p ↓- PGK1↑	
PCa	circ_0086722	Cell line/Tissue	Prognostic	↑	miR-339-5p	circ_0086722↑-miR-339-5p↓-STAT5A↑	[149]
PCa	circDDIT4	Tissues and serum samples	Diagnostic	↑	ELAVL1/HuR	circDDIT4↑-ELAVL1/HuR↓ -ANO7↓	[150]
PCa							
OC	circWHSC1	Tissue	Prognostic	↑	miR-145 and miR-1182	circWHSC1↑- miR-145 and miR-1182↓-MUC1 and hTERT↑	[151]
OC	circPLEKHM3	Tissue	Prognostic	↓	miR-9	circPLEKHM3↓-miR-9↓ AKT1↓	[152]
BCa	cTFRC	Tissue	Prognostic	↑	miR-107	cTFRC↑- miR-107↓- TFRC↑	[153]
BCa	circMTO1	Tissue	Prognostic	↑	miR-221	–	[154]
Osteosarcoma	circPVT1	Serum	Diagnostic	↑	–	–	[155]
Glioma	circ_0034642	Tissue	Prognostic	↑	miR-1205	circ_0034642↑-miR-1205↓-BATF3↑	[156]
Nasopharyngeal carcinoma	CDR1as	Biopsy	Prognostic	↑	miR-7-5p	CDR1as↑-MiR-7-5P↓-E2F3↑	[157]

*RCC* renal cell carcinoma, *LC* lung cancer, *GC* gastric cancer, *CRC* colorectal cancer, *BC* breast cancer, *HCC* hepatocellular carcinoma, *PC* pancreatic cancer, *OC* ovarian cancer, *NSCLC* non-small cell lung cancer, *BCa* bladder cancer, *SF3B3* splicing factor 3b subunit 3, *PI3K* phosphoinositide 3-kinase, *NER* nucleotide excision repair, *MMP1* matrix metalloproteinase 1, *MMP17* matrix metalloproteinase 17, *HMGB3* high-mobility group box 3, *FAM83A* family with sequence similarity 83 member A, *ERK1* extracellular signal-regulated kinase 1, *PTEN* phosphatase and tensin homolog, *Bcl-2* B-cell lymphoma 2 protein, *TMEM14A* transmembrane protein 14A, *GNB1* guanine nucleotide-binding protein subunit beta-1, *EGFR* epidermal growth factor receptor, *PIK3CD* phosphatidylinositol 3-kinase catalytic subunit delta, *PPDPF* protein phosphatase 1D magnesium-dependent delta isozyme, *MACC1* metastasis-associated in colon cancer 1, *ABCC1* ATP-binding cassette subfamily C member 1, *LAD1* ladinin 1, *ZNF281* zinc finger protein 281, *LATS1* large tumor suppressor kinase 1, *FOXO3* forkhead box O3, *CBX8* chromebox protein homolog 8, *YAP* yes-associated protein, *HMGB1* high-mobility group box 1, *RAGE* receptor for advanced glycation end products, *MTSS1* metastasis suppressor 1, *TFRC* transferrin receptor, *BATF3* basic leucine zipper ATF-like transcription factor 3.

Overall, developing novel, non-invasive, extremely precise, and accurate biomarkers for early cancer screening, diagnosis, therapy, and prognosis monitoring is necessary.

### 4.2 CircRNAs as inhibitors in cancer therapy

Almost every human malignancy has circRNAs that are involved in its pathogenesis. In our previous discussion, we established that circRNAs compete with target genes for miRNA-binding sites, allowing them to regulate gene expression in a manner similar to that of miRNA sponges [158]. Current research has shed light on how circRNAs, which function as miRNA inhibitors, regulate the migration and prognosis of cancer cells. For instance, circ-ZKSCAN1 inhibits BC progression by miR-1178-3p sponging through the miR-1178-3p/p21 axis and serves as a predictive factor for recurrence [159]. Similarly, Zhang et al. demonstrated that circRNA-104075 can inhibit miRNA-104075 in HCC tissue and serve as a new diagnostic biomarker [138]. Therefore, targeting circ 104075 might lead to novel HCC diagnosis and therapy approaches.

### 4.3 CircRNAs as therapeutic targets in cancer therapy

In the area of therapeutic targets, tumor-promoting, and tumor-suppressive circRNAs have the potential to be used as therapeutic targets in cancer. CircRNAs have a unique back-splicing junction sequence that allows them to be targeted selectively without modifying the parent mRNA. As a result, carcinogenic circRNAs can be a target for siRNAs and undergo Ago2-mediated destruction [160]. In contrast, tumor-suppressing circRNAs may be expressed ectopically utilizing expression vectors with lengthy reverse complementary sequences that allow the enhanced sequence to circulate [161]. A potential therapeutic approach could be to target circRNAs or associated pathways, as their dysregulation can lead to cancer.

Several biomolecules, such as proteins, shRNA, and siRNA, inhibit or enhance circRNAs. For instance, Fang et al. demonstrate that circAGO2 is enhanced with HuR protein and overexpressed in several malignant tissue samples. Stimulation of circAGO2 led to boosted growth, invasion, and metastasis in cell culture and animal models [162]. In contrast to stimulation, the inhibition of circAGO2 by shRNA spurred tumor growth in vivo since circAGO2 activity may be controlled by the inhibition of circAGO2 and HuR response; this can be accomplished with the assistance of a HuR suppressor peptide delivery [163]. Moreover, Zhang et al. investigated that circCUX1 could significantly be overexpressed and enhanced tumor cell growth, invasion, and migration by inhibiting miR-16-5p in a neuroblastoma sample, while inhibited circCUX1 with shRNA lowered tumor proliferation in a mouse model [164].

In addition, antisense oligonucleotides have also been applied to downregulate circular RNAs. For example, in colorectal cancer (CRC), the connection between circLONP2 and DDX1 led to the overexpression of cells that produce metastases, which facilitated invasion and propagation. Conversely, the in vivo downregulation of circLONP2 by antisense oligonucleotides prevented CRC metastases [165]. Furthermore, in patients with acute myeloid leukemia, the expression of circ_0004277 was reduced after chemotherapy treatment but increased again when patients relapsed after full remission, demonstrating the relationship between upregulation and treatment success [166].

Moreover, according to the findings of the study conducted by Fang and his colleagues, it was proposed that the administration of FUS-medicated circ_0002099 might potentially enhance the EMT process in the progression of BLCA by means of the miR-217-5p/miR-103a-3p/KRAS axis-WNT/β-catenin axis. Additionally, it has the potential to serve as a therapeutic target and a promising predictive biomarker for BLCA [167]. Similarly, CiRS-7 is a promising biomarker for predicting the prognosis of CRC patients, and it has the potential to be used as a therapeutic target for lowering the activity of EGFR-RAF1 in CRC patients [168]. Additionally, in a patient-derived CRC xenograft model, Yang and his team demonstrated that the injection of shRNA into the tail vein precisely targets circPTK2 and reduces the spread of tumor metastases. Thus, they identified that circPTK2 is a promising biomarker for early diagnosis of CRC metastasis and provides a potential therapeutic target for CRC [169].

Recently, there are developing in clinical research that are focusing on various elements of circRNAs as therapeutic targets in cancer therapy. These studies are focusing on circRNA expression patterns, functional roles, and therapeutic interventions [170,171]. For example, in ovarian cancer, Zhu et al. demonstrated that circNUP50 was increased in DDP-resistant OC cells [172]. This would allow for the formation of platinum-resistant conditions in OC by promoting cell proliferation, changing the cell cycle, and reducing apoptosis. Thus, they proposed that circNUP50 is a new therapeutic target that modifies p53 ubiquitination to induce cisplatin resistance in OC.

According to these results, the regulation of circular RNAs is not constant and varies with the severity of diseases, supporting the potential therapeutic roles of circRNAs in cancer treatment. Table 2 explains the mechanism of action and research sources for the various cancer types where circRNAs can be used as therapeutic targets.

**Table 2:** CircRNAs as therapeutic targets in cancer.

Type of cancer	circRNAs	Source	Regulation	miRNA sponge	Mechanism	Ref.
LC	circUBAP2	Clinical sample, cell line	↑	miR-3182	circUBAP2↑-miR −3182↓- KLF4↑	[173]
LC	circRNA_102231	Tissue	↑	–	Unknown	[68]
LC	hsa_circ_100395	Tissue	↓	miR-1228	hsa_circ_100395↓-miR −1228↑- TCF21↓	[174]
LC	circFADS2	Tissue, cell line	↑	miR-498	circFADS2↑-miR-498↓- HMGA2↑	[175]
LC	circRNA-FOXO3	NSCLC specimen	↓	miR −155	circRNA-FOXO3↓-miR −155↑-FOXO3↓	[176]
LC	circCDYL	NSCLC tissue, plasma, cell line	↑	miR-185- 5p	circCDYL↓-miR-185- 5p↑/TNRC6A↓-ERK1/2↑	[177]
LC	circSEC31A	–	↑	miR −376a	circSEC31A↑-miR −376a↓-SEC31A↑	[178]
LC	hsa_circ_0020123	Tissue	↑	miR-144	hsa_circ_0020123↑- miR-144↓-ZEB1↑/EZH2↑	[179]
LC	circ_0003998	–	↑	miR136-5p	circ_0003998↑- miR136-5p↓-CORO1C	[180]
LC	circCCDC66	NSCLC cell line, human bronchial epithelial cell line	↑	miR −33a-5p	circCCDC66↑-miR −33a-5p↓-KPNA4↑	[181]
LC	hsa_circ_0002874	Cell line	↑	miR-1273f	hsa_circ_0002874↑- miR-1273f↓-MDM2/P53↑	[182]
LC	hsa_circ_11780	NSCLC tissue, cell line	↓	miR-544a	hsa_circ_11780↓-miR −544a↑-FBXW7↓	[183]
LC	hsa_circ_0038646	Cells, tissue	↑	miR-331-3p	hsa_circ_0038646↑-miR-331-3p↓-GRIK3↑	[184]
LC	hsa_circ_0010235	NSCLC tissue, cell line	↑	miR-433-3p	hsa_circ_0010235↑-miR-433-3p↓-TIPRL↑	[185]
LC	hsa_circ_0001073	Lung cancer cells	↓	miR-626	hsa_circ_0001073↓-miR-626↑-LIFR↓	[186]
LC	circRNA_103993	NSCLC cells, human bronchial epithelial cell line	↑	miR-1271	circRNA_103993↑-miR −1271↓- ERG↑	[187]
LC	circP4HB	NSCLC tissue, cell line	↑	miR-133a	circP4HB↑-miR-133a5p↓- vimentin↑	[188]
LC	circARHGAP10	NSCLC tissue, cell line	↑	miR-150-5p	circARHGAP10↑-miR −150-5p↓-GLUT1↑	[189]
LC	circVANGL1	NSCLC tissue, cell line	↑	miR-195	circVANGL1↑-miR −195↓-Bcl-2↑	[190]
BC	CircDENND4C	Tissue, cell line	↑	miR-200b, miR-200c	Functions in low-oxygen environments as a miR-200b and miR-200c sponge.	[191,192]
BC	CircFBXW7	Cell line	↓	miR-197-3p	Sponging miR-197-3p upregulates FBWX7 expression.	[193]
BC	CircTADA2A	Tissue, cell line	↓	miR-203a-3p	Activates miR-203a-3p, which in turn increases cytokine signaling regulator SOCS3 expression.	[194]
BC	CircWWC3	Tissue	↓	miR-26b-3p, miR-660-3p	Increases expression of EGFR, GRB2, PAK4, MAPK1, and AKT1 while acting as a sponge for miR-26b-3p and miR-660-3p (Ras signaling pathway)	[195]
BC	CircRNF20	Tissue, cell line	↑	miR-487a	Suppresses miR-487a, which targets HIF1α′s 3 UTR.	[196]
BC	Circ-CDYL	Tissue	↑	miR-1275	targets ULK1 and ATG7 mRNAs linked to autophagy and autophagosomes, acting as a miR-1275 mimic.	[197]
CRC	circACAP2	Tissue, cell line	↑	miR-21-5p	T lymphoma invasion and metastasis protein 1 expression was suppressed by circACAP2 silencing, which increased the expression of miR-21-5p.	[198]
CRC	hsa_circ_0020397	CRC cells	↑	miR-138	The expression of the miR-138 targets TERT and PD-L1 showed that has circ 0020397 did reduce miR-138 activity.	[199]
PCa	circABCC4	PCa cells and tissue	↑	IGF2BP2	circABCC4↑ -IGF2BP2↑-CCAR1↑	[200]
PCa	circRBM33	PCa cells and tissue	↑	ARSI	circRBM33↑- ARSI↓-PDHA1↑	[201]
CRC	hsa_circ_0055625	Tissue	↑	miR-106b-5p	circ_0055625↑-miR-106b-5p (miR-106b) ↓-ITGB8↑	[202]
CRC	hsa_circ_0000523	Tissue, cell line	↓	miR-31	hsa-circ-0000523↓- miR-31↑-Wnt/β-catenin signaling pathway↑	[203]
CRC	has_circ_103809	Tissue, cell line	↓	miR-532-3P	hsa_circRNA_103809↓-miR-532–3p↑-FOXO4 axis↑	[204]
RCC	circPUM1	Tissue	↑	miR-340-5p	circPUM1↑-miR-340-5p↓-FABP7↑	[39]
RCC	circ_0005875	Tissue	↑	miR-502-5p	circ-0005875↑-miR-502-5p↓-ETS1↑	[205]
RCC	hsa_circ_0054537	Tissue	↑	miR-130a-3p	hsa_circ_0054537↑- miR-130a-3p↓-cMet↑	[206]
RCC	circPTCH1	Tissue, cell line	↑	miR-485-5p	circPTCH1↑- miR-485-5p↓MMP14 and EMT↑	[207]

*LC* lung cancer, *BC* breast cancer, *CRC* colorectal cancer, *RCC* renal cell carcinoma, *NSCLS* non-small cell lung cancer, *KLF4* kruppel-like factor 4, *TCF21* transcription factor 21, *HMGA2* high mobility group at-hook 2, *FOXO3* forkhead box o-3, *TNRC6A* trinucleotide repeat containing adaptor 6A, *ERK1* extracellular signal regulated kinase 1, *SEC31A* secretory pathway component 31A, *ZEB1* zinc finger E-Box binding homeobox 1, *EZH2* enhancer of zeste homolog 2, *KPNA4* karyopherin alpha 4, *MDM2* mouse double minute 2, *P53* protein 53, *FBXW7* F-box/WD repeat containing protein 7, *GRIK3* glutamate receptor ionotropic kainite 3, *LIFR* leukemia inhibitory factor receptor, *GLUT1* glucose transporter 1, *Bcl-2* B-cell lymphoma 2, *SOCS3* suppressor cytokine signaling 3, *EGFR* epidermal growth factor receptor, *GRB2* growth factor receptor bound protein 2, *PAK4* p21-activated kinase 4, *MAPK1* mitogen-activated protein kinase 1, *AKT1* protein kinase B, *HIF1* α hypoxia inducible factor, *ULK1* unc-51 like autophagy activating kinase 1, *ATG7* autophagy-related 7, *TERT* telomerase reverse transcription, *PD-L1* programmed cell death ligand 1, *ITGB8* integrin subunit beta 8, *FOXO4* forkhead box 4, *FABP7* fatty acid binding protein 7, *ETS1* E26 transformation-spesific-1, *MMP14* matrix metallopeptidase 14, *EMT* epithelial mesenchymal transition.

### 4.4 CircRNAs as a vaccine for cancer therapy

Recently, artificial circRNA has been created to discover how they might be used as a novel class of mRNA therapeutics and vaccines. According to several studies, since regular mRNAs were translated to function as antigens, circRNAs can be used to express proteins [12]. Due to this, circRNAs and mRNAs are suitable candidates to produce vaccines. For instance, novel clinical research has demonstrated that the translation of proteins could be achieved by introducing mRNAs into the dendritic cells to be used as antigen-producing molecules to create vaccines for cancer immunotherapy [208]. This makes it possible to translate circRNAs into proteins, which would then generate functional antigens and activate CD8^+^ T cells to develop vaccines from cancer stem cells. According to Sun et al., mature DCs with CD133+ hepatocellular carcinoma stem cell RNA can trigger vitro cytotoxic T lymphocyte responses against hepatocellular cancer stem cells [209]. Furthermore, when 9L glioma stem-like cell RNA was injected into dendritic cells, the tumor's growth was dramatically inhibited, and the survival rate of rats with gliomas increased [210].

Likewise, circRNAs can act as tumor antigens or vaccine adjuvants that stimulate innate and adaptive immune responses. According to Chen et al., the intracellular sensor retinoic-acid-inducible gene-I regulated the immune response to exogenous circRNAs, and transfecting HeLa cells in vitro to produce circRNA may trigger innate immunity gene expression [211]. Exogenous unmodified circRNA adjuvant might trigger antigen-specific T and B cell responses by interacting with and activating RIG-1 [212]. Li et al. showed that the RNA vaccine's stability was effectively increased by circularizing linear RNA molecules to produce stable circRNA molecules with long-lasting protein expression capabilities. Thus, by covering the antigen-coding circRNA in LNP to facilitate in vivo synthesis, scientists created a novel circRNA vaccination platform. This approach demonstrated superior anti-tumor effectiveness in many animal tumor models and was able to induce substantial innate and adaptive immune activation [12].

Despite the apparent benefits of circRNAs acting as novel vaccines, circRNA investigations are still in their early stages; hence, all relevant clinical trials have concentrated on mRNA vaccines. Additionally, recombinant enzymes are still used to create circRNAs in vitro, which is expensive if circRNAs are needed for vaccines [213]. Thus, research on this model is necessary to clarify circRNA's application as a vaccine for cancer therapy.

## 5 Challenges of the use of circular RNA as a cancer therapy and strategies to overcome

**Fig. 4:**
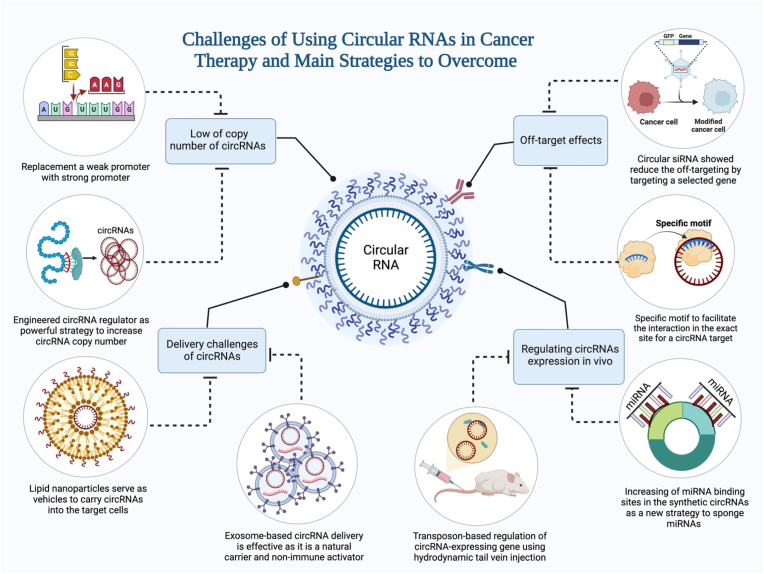
The primary obstacles to employing circRNAs as therapeutic biomolecules and the methods to overcome them so that they can be utilized in cancer treatment as a potential replacement for traditional therapeutic biomolecules, which are currently less effective against most types of cancer.

Although the previous description shows that circRNA is an effective technique, its discovery and implementation in human tumors make it a targeting method with various limits and concerns that make its use in clinical trials challenging. Low circRNA copy number, delivery method, controlling circRNA expression in vivo, and off-targeting are the significant concerns with circRNAs that have been identified (Fig. 4).

### 5.1 Low copy number of circRNA

One of the most crucial metrics to shed light on the efficacy of circRNAs in cancer treatment is the quantity of circular RNA. If circRNAs are to be utilized as successful treatment strategies, it is crucial to identify their precise functional mechanism; however, this may be more challenging due to the low copy number of circRNA. For example, if a circRNA is numerous and contains a large number of miRNA binding sites, it may be able to function as an effective miRNA sponge [214]. However, if a circRNA has a low copy number, its sponging efficacy would be limited even if it has many miRNA binding sites. Similarly, a circRNA with a high abundance but few miRNA binding sites would have low sponging efficiency and is improbable to serve as a miRNA sponge [7]. Therefore, the copy quantity of circRNAs must be ascertained to conclude that they can act via protein binding or other processes such as miRNA sponging. The initial stage of circRNA-based therapeutic approaches should be considered the inadequate quantity of circRNA.

#### 5.1.1 Strategy to overcome the low copy number of circRNA

CircRNA levels can be altered in particular tissues, organs, and animal systems [215]. An appropriate cancer therapy requires upregulating a specific gene expression through miRNA sponging, which depends on circRNA and miRNA interaction. For this reason, the circRNA to miRNA ratio should be relatively high as it can enhance the sponging of miRNA [7]. If this ratio is high, it shows the efficacy of circRNA as a molecule that can address gene dysregulation in cancer disease. However, a low ratio makes clinical applications less efficient or inefficient [7].

**Fig. 5:**
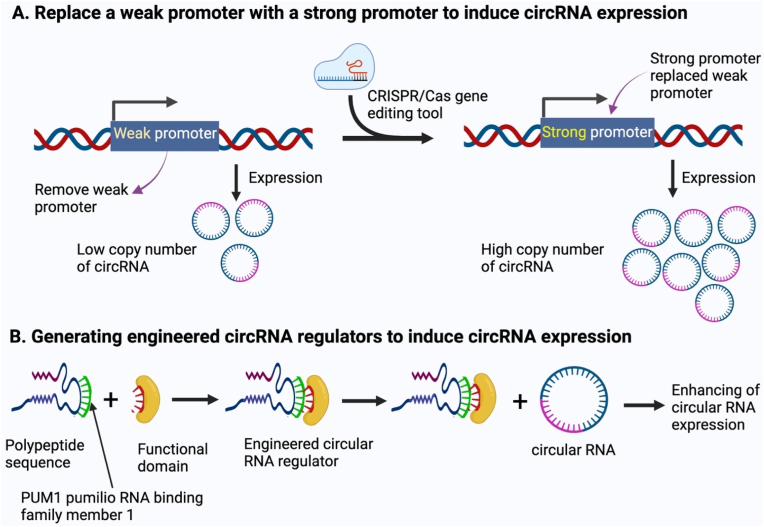
shows the two strategies for increasing the copy number of circRNAs when used to treat cancer cells. The strategies are (A) replacing a weak promoter with a strong promoter by applying gene editing tools such as CRISPR/Cas technology to induce circRNA expression and (B) generating engineered circRNA regulators by using a gene encoding a member of the PUF family to induce circRNAs.

Genetic manipulation in cancer therapy has been significantly enhanced with the development of genome editing tools such as ZFN, TALEN, and CRISPR-Cas programs. As illustrated, CRISPR‐Cas9 has made several genetic applications possible, like promoter editing for generating quantitative trait variation and its widespread use in knocking out protein-coding genes [216] (Fig. 5).

### 5.2 Delivery challenges of circRNA in cancer therapy

Delivery of therapeutic agents is a fundamental and essential step in cancer therapy, and any limitation in this system leads to a decrease in the efficiency of the cancer therapy strategy. In cancer therapy, the delivery of therapeutic molecules is challenging because of the increase in immunological activation, bio-incompatibility, and ineffectiveness [217,218]. Several practical and trustworthy techniques can address this limitation, such as lipid nanoparticle or exosome-based delivery.

#### 5.2.1 Strategies to overcome challenges of circRNA delivery in cancer therapy

Many approaches and strategies have been proposed, but unfortunately, the growing new side effects limit their delivery efficiency in the treatment of cancer. Here, we demonstrate two feasible techniques that enable therapeutic approaches to work efficiently.

#### 5.2.2 Lipid nanoparticle-based delivery of circRNA

**Fig. 6:**
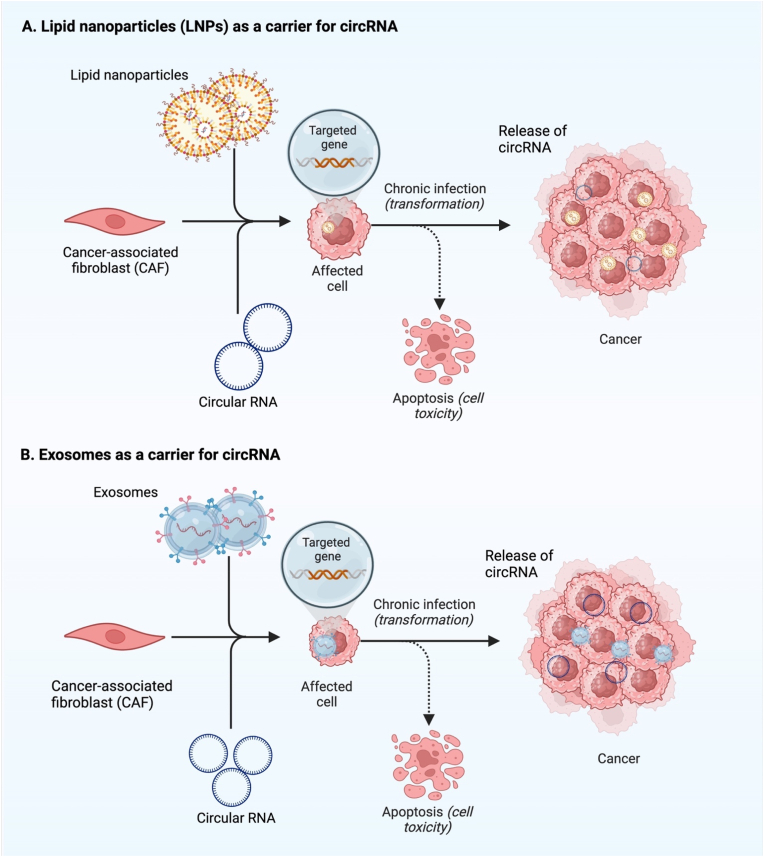
Highlights the two practical approaches to address the limitations of the delivery method in delivering circRNAs for cancer treatment. (A) Lipid nanoparticles (LNPs) and (B) exosomes, both of which are carriers for circRNAs.

Nanoparticles can carry drugs and deliver them to therapeutic targets [219], and their potential uses in therapeutic medications, molecular delivery imaging, and drug combinations that combine diagnostic and therapeutic properties are actively being investigated. To target specific cells with either endogenous or foreign ligands, lipid nanoparticles (LNPs) are the most advanced nanoparticle carriers of small interfering RNA (siRNA), messenger RNA (mRNA), and circular RNA (circRNA) [220]. LNPs are nanoparticles that can deliver medications to disease areas for enhanced therapy efficacy [221]. In response to either internal or external signals, they can encapsulate circRNA and transport it to a targeted cell population; however, upon uptake, their membranes can become unstable, enabling circRNA to escape into the cytosol and reach their targets [220]. Li et al. demonstrated their successful and efficient use of lipid nanoparticles to encapsulate and deliver circRNA into the aggressive tumor in mice in a trial [12]. Several potential applications for these nanoparticles are currently under study, including delivering diagnostic and therapeutic compounds [222]. Based on the results of these studies, lipid nanoparticles show significant promise as a delivery mechanism for circRNA-targeting therapeutics (Fig. 6a).

#### 5.2.3 Gold nanoparticles (AuNPs) delivery

The use of nanoparticles as a delivery mechanism has significantly boosted the viability of circRNA-based therapeutics in vivo. Several studies have shown that gold nanoparticles (AuNPs) are a reliable delivery strategy in animals due to their great stability, purity, and easily adaptable surface [223]. For instance, in order to target the binding sites on circDnmt1 for Auf1 and p53 proteins, Du et al. administered AuNPs coupled with siRNA as a breast cancer therapeutic approach [224]. In addition, tumor growth was suppressed, and mouse survivability was prolonged by AuNP delivery of AONs inhibiting binding sites on circCcnb1 for Ccnb1 and Cdk1 [225]. According to this research, circRNA-targeting medicines may find a useful delivery method in nanoparticle form.

Moreover, circRNA expression plasmids have also been delivered in vivo via nanoparticles. Lu et al. found that the distribution of circEHMT1 plasmids via nanoparticles prevented the spread of breast cancer to the lungs in mice [226]. Similarly, the delivery of circFoxo3 plasmids with AuNP led to an increase in the rate of apoptosis in tumor cells and a reduction in the development of tumors [227].

Even though AuNPs effectively deliver circRNA targets in animal models, their safety in clinical settings remains largely unknown. Previous research has shown that AuNPs' hazardous effects are dependent on their size, with smaller AuNPs having more harmful effects [228]. As a result, AuNPs' characteristics can be adjusted to satisfy safety standards, offering circRNA-targeted medication delivery systems a bright future.

#### 5.2.4 Exosome-based delivery of circRNA

Exosomes, also known as extracellular vesicles (EcVs), can be found in nearly every cell, tissue, and bodily fluid. In disease pathobiology, they aid in intercellular signaling and keep tissues in a state of homeostasis [229,230]. Exosomal cargo contains 9769 proteins, 2838 microRNAs, 3408 messenger RNAs, and 1116 lipids, according to research [231].

Exosomes hold great promise as a therapeutic tool for various illnesses, including cancer, because they can effectively move tiny chemicals between cells [232]. Exosomes can be utilized to deliver treatments to cancer cells because of their unique characteristics, including their nano-size, double lipid membrane, capacity to serve as numerous carriers, good histocompatibility, high bioavailability, minimal cytotoxicity, and immunogenicity [233]. They can deliver a wide range of molecules, such as small interfering circRNAs (circRNAs) [4], microRNAs (miRNAs) [234], proteins [235], lipids [236], and DNA fragments [237] in their native state. Numerous studies have discussed the potential use of circRNAs as novel therapeutic targets and biomarkers in treating various illnesses, including cancer. Exo-circRNAs are circRNAs found in exosomes that can be found to support conventional diagnostic techniques and used to suppress the spread of malignancy [238].

Exosomal circRNA is taken by surrounding or distant cells and alters numerous physiological and pathological states, potentially boosting cell communications and spreading cancer. For the first time, in 2015, Li et al. discovered that exosomes are a rich source of circRNAs, and they were shown to be abundant in exosomes compared to parental cells, according to genome-wide RNA-seq investigations [239]. CircRNA sorting to exosomes can be controlled by adjusting parental cell levels of associated miRNA. This, in turn, can influence the biological activity of target cells, which are more stable and tolerant to their environment [240] (Fig. 6b).

This confirmed delivery ability of exosomes as carriers for circRNAs for cell-to-cell communication makes the exosomes more reliable and feasible carriers for circRNA delivery in cancer therapy-based circRNA strategy.

### 5.3 Regulating circRNA expression in vivo

To apply the effective therapeutic role of circRNAs in cancer, there should be approaches to regulate circRNA expression in vivo. Here, it can be concluded that new effective strategies can overcome the limitations of regulating circRNA expression in vivo.

#### 5.3.1 Strategies to overcome regulating circRNA expression in vivo

**Fig. 7:**
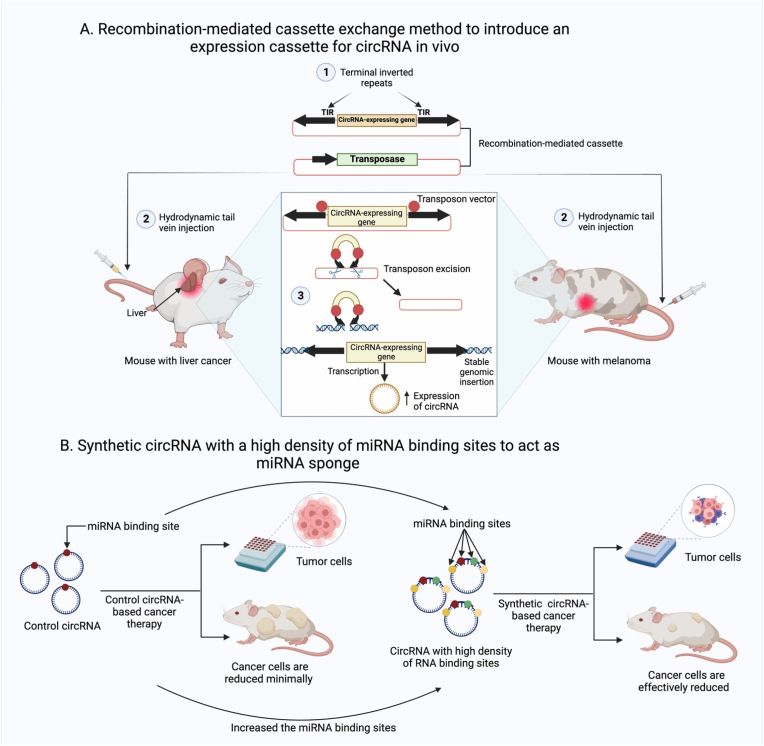
Regulation of circRNA expression in vivo using new genetic methods for long-term production of potential circRNAs. Transposons are employed to deliver a circRNA production cassette to the livers of mice by hydrodynamic tail vein injections. At the same time, the recombination-mediated cassette exchange method is used to introduce an expression cassette for the circRNA into the mouse genome.

Innovative genetic approaches for the sustained synthesis of prospective circRNAs hold great promise for controlling circRNA expression in vivo. For instance, transposons were employed to deliver a circRNA production cassette to the liver of mice by hydrodynamic tail vein injections (Fig. 7a). Mecozzi et al. inserted an expression cassette encoding the circRNA into the mouse genome using the recombination-mediated cassette exchange technique. Consequently, every mouse genome produces ectopic circRNA [241]. Further, to specifically promote circRNA expression in melanoma cells, researchers created a circRNA-expressing animal model of the disease using the same strategy [241]. These new methods and tools for functional analysis will alleviate some of the restrictions currently associated with circRNA expression systems.

Furthermore, circRNAs can be synthesized to increase their expression and functionality. The capacity of circRNA to act as a miRNA sponge is affected not only by the number of copies but also by the number of miRNA binding sites. To effectively sponge miRNAs, a synthetic circRNA with a high density of miRNA binding sites can be designed [[242], [243], [244]]. Therapeutic interventions may be possible through this regulatory mechanism, which also has significance for gene expression (Fig. 7b).

**Fig. 8:**
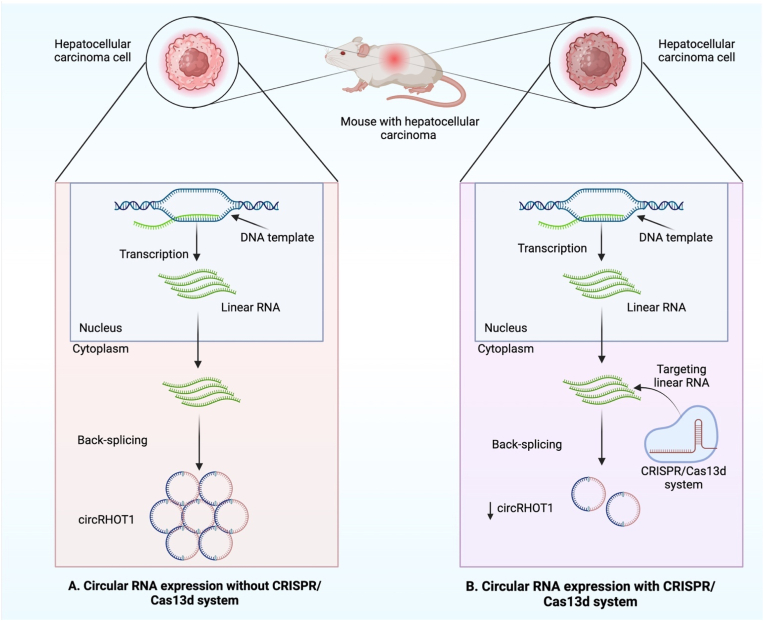
Illustrates the expression of circular RNAs in an animal mouse model. (A) Overexpression of circRNA in mouse model hepatocellular carcinoma without CRISPR/Cas13. (B) Through the depletion of complementary sequences, the CRISPR/Cas13d technique-induced knockdown of oncogenic circRHOT1 in a mouse model inhibits the growth of HCC cells.

Another new technology, clustered regularly interspaced short palindromic repeats (CRISPR), has entered the list of optimizers for circRNA regulation in vivo. Gao et al. used RNA-targeting type VI CRISPR effector RfxCas13 to suppress circRNA expression at the RNA level [245]. Similarly, Zhang et al. improved the strategy for creating CRISPR/Cas13 gRNAs with the ideal specificity and efficiency for reducing circRNA expression [246]. Additionally, Wang et al. explored essential circRNAs using optimized Cas13d technology and reported that knocking down of circRHOT1 leads to inhibition of HCC cell proliferation by depleting complementary sequences (CSs) [247] (Fig. 8).

Based on the aforementioned successful studies that control circRNA expression in vivo, this novel approach could propel circRNA-based cancer treatments forward and make them more viable. This will pave the way for other applications and the start of clinical trials.

### 5.4 Off-target effects in non-cancerous cells and tissues

While significant progress has been made in circRNA regulation in vivo, much more needs to be done before these technologies may be applied in clinical settings [248,249]. One obstacle is that a single circRNA molecule can function in a variety of tissues and diseases, for example, abnormal brain development [250], Parkinson's disease [251], myocardial infarction [252], diabetes [253], and femoral head necrosis [254], all have links with CDR1as. Similarly, circFoxo3 is implicated in the regulation of aging in the heart [255], as well as chemical resistance in prostate cancer [256]. Thus, using circRNA expression as a target for cancer therapy may have unexpected, adverse effects on other tissues or organs. To be a viable therapeutic option, circRNA-based therapies also need to increase therapeutic specificity and reduce off-target effects on other tissues or organs.

#### 5.4.1 Strategies to overcome the circRNAs off-targeting effects

The base of the incorrect targeting in circRNA-based cancer therapy lacks a specific motif to facilitate the interaction in the exact site for a circRNA target. HEK293A cells were transfected with circular siRNAs targeting GFP or luciferase reporter genes with a circular sense and linear antisense strand. This allowed the siRNAs with the circular structural motif to effectively block the effects of sense strand RNA-induced off-target gene expression [257]. This discovery was made in response to the need to address the issue of circRNA off-targeting.

Circular siRNAs significantly reduced the off-target effect brought on by the sense strand of siRNA, as demonstrated by using RenillaXas, a rebuilt Renilla luciferase reporter gene. This new approach is significantly functional in reducing off-targeting in circRNA-based cancer therapy [257]. Moreover, off-target effects can be reduced to a minimum through careful design of circRNA sequences. In order to improve the specificity of circRNAs and decrease the amount of non-specific binding to undesired targets, it is possible to optimize their length, secondary structure, and sequence composition [258]. Although, the stability and specificity of circRNAs can be improved through the introduction of chemical modifications, such as 2′-O-methyl or locked nucleic acid modifications. These alterations have the potential to either increase the affinity of circRNA for binding to target molecules or decrease the affinity for binding to non-specific targets [259,260]. Further, through the incorporation of cell-specific promoters into circRNA expression constructs, it is possible to restrict circRNA production to particular cell types, hence decreasing the impact of off-target expression in cells that are not the target. The utilization of this technique guarantees that circRNAs are preferentially expressed in the cell populations that are wanted, hence boosting therapeutic efficacy while simultaneously limiting off-target consequences [261]. Likewise, off-target effects can be reduced by selecting vector systems that are suitable for the delivery of circRNA. It is possible to increase circRNA localization and prevent unwanted interactions with off-target molecules by selecting vectors that have a high transfection efficiency and specificity for the target cell [262,263].

On the other hand, the CRISPR/Cas system is another developing and powerful strategy can be used to reduce off-target effects. For instance, using a machine learning methodology, Cheng et al. discovered that DeepCas13 effectively differentiates efficient sgRNAs from inefficient ones, and Cas13d′s effect is linked to the guide's on-target cleavage effect in hepatocellular carcinoma [264]. Moreover, the CRISPR-dCas9 and CRISPR-Cas9 systems can also be used to demonstrate the function of this circular gRNA in vitro. Researchers have proposed a novel design for gRNA that might promote rapid circular gRNA assembly inside cells. The twister ribozymes are positioned on either side of the gRNA of interest in this circular pattern. It demonstrated a striking decrease in the off-target rate, together with decreased efficiency [265]. Furthermore, comparative investigations indicated that RfxCas13-mediated circRNA knockdown exhibited significantly lower off-target effects on cognate mRNAs than shRNA/siRNA-mediated knockdown in mouse embryos [266]. Thus, the off-targeting effects of circRNAs during their usage in cancer therapy could be avoided or limited if the strategies listed above are used.

### 5.5 CircRNA-lncRNA sponge ceRNA interaction

Cancer study is greatly impacted by the complex mechanisms underlying the interactions between different ncRNAs, including lncRNAs and circRNAs. LncRNAs and circRNAs can interact and sponge miRNAs, which affects regular physiological functions and controls mRNA expression and, in turn, cell physiological state [267]. However, the significant implications for cancer may result from complex interactions between circRNAs and lncRNAs and brings therapeutic limitation. For instance, one of the primary mechanisms involves sponge regulation, where lncRNAs act as sponges for circRNAs or vice versa. These interactions can regulate gene expression precisely, which plays a role in the initiation and spread of cancer cells by sequestering miRNAs or other RNA-binding proteins [268].

#### 5.5.1 Strategies to overcome CircRNA-lncRNA interaction

The interplay between circRNAs and lncRNAs presents several challenges in the context of cancer therapy strategies. Creating synthetic RNA molecules that mimic the miRNA binding sites on the circRNAs or lncRNAs that mediate their interaction is one alternative [268]. These synthetic molecules may competitively bind to lncRNAs, preventing their interaction with the circRNA and disrupting the ceRNA regulatory network. Additionally, CRISPR/Cas-based genome editing technologies offer precision in targeting and disrupting the ceRNA interaction at the genetic level [269]. CRISPR/Cas systems can minimize the ceRNA effect by altering circRNA-lncRNA binding sequences, breaking the ceRNA connection. Further, accurate analysis and prediction of these interactions depend on sophisticated computer algorithms and bioinformatics tools. It is essential to achieve both specificity and selectivity when addressing circRNA-lncRNA sponge interactions to prevent any interference with regular cellular processes. Enhancing specificity can be accomplished by techniques such creating tiny molecules or altered oligonucleotides that bind to target ceRNAs only while sparing non-targeted RNAs [270].

## 6 Conclusion and future perspectives

CircRNAs are new RNAs recently found in numerous species by high-throughput sequencing. CircRNAs play critical roles in cancer therapy and have been identified as possible attractive non-coding molecules. Their structural stability and resistance to RNase enzymes make them promising diagnostics and therapeutic options.

CircRNAs are abundant in tumors; they bind to proteins, control transcription, and translation, and act as sponges for miRNAs. Therefore, cirRNAs have applications in prognosis, drug-delivery design for target therapy, therapeutic response, and early diagnosis. Their remarkable properties and powerful functions have attracted the attention of researchers and clinicians in their study and potential therapeutic applications.

Despite advanced studies, several open concerns remain about the clinical use of circRNAs in cancer therapy. This study highlights various novel developments and therapeutic possibilities of circRNA-based cancer therapy. These include the utilization of circRNAs as a diagnostic and prognostic biomarker in cancer patients, the use of adjuvant-based vaccines with sponge oncogenic miRNAs, and the potential use of carcinogenic circRNAs as a therapeutic target for siRNAs. Further, it explores the main challenges of using circular RNA as a cancer therapy, like low copy numbers of circRNAs, delivery challenges, regulation expression, and off-targeting effects. We also highlighted the primary strategies to overcome these limitations.

Future studies should further investigate the efficacy and safety of using circRNA-based therapies. Critically, in vivo research is necessary to determine whether CRISPR/Cas technology is viable for efficient and specifically knocking down circRNAs. We assume that future discussions will revolve around methods to overcome the primary obstacles to using circRNAs in place of conventional cancer treatments and that additional research will be necessary to confirm the therapeutic efficacy of these molecules.

## Ethics approval and consent to participant

Not applicable.

## Consent of publication

Not applicable.

## Availability of data and materials

Not applicable.

## CRediT authorship contribution statement

**Goran Sedeeq Hama Faraj:** Methodology, Investigation. **Bashdar Mahmud Hussen:** Methodology, Investigation. **Snur Rasool Abdullah:** Methodology, Investigation. **Mohammed Fatih Rasul:** Resources, Investigation. **Yasaman Hajiesmaeili:** Methodology, Investigation. **Aria Baniahmad:** Methodology, Investigation. **Mohammad Taheri:** Writing – original draft, Supervision, Investigation.

## Declaration of competing interest

The authors declare they have no conflict of interest.

## Abbreviations

ATG7: Autophagy-related 7
BC: Breast cancer
CRC: Colorectal cancer
EGFR: Epidermal growth factor receptor
EMT: Epithelial-mesenchymal transition
ERK1: Extracellular signal-regulated kinase 1
EZH2: Enhancer of Zeste homolog 2
FABP7: Fatty acid binding protein 7
FOXO3: Forkhead box o-3
FOXO4: Forkhead box 4
GLUT1: Glucose transporter 1
GRB2: Growth factor receptor bound protein 2
GRIK3: Glutamate receptor ionotropic kainite 3
HMGA2: High mobility group at-hook 2
ITGB8: Integrin subunit beta 8
KLF4: Kruppel-like factor 4
KPNA4: Karyopherin alpha 4
LC: Lung cancer
LIFR: Leukemia inhibitory factor receptor
MAPK1: Mitogen-activated protein kinase 1
MDM2: Mouse double minute 2
MMP14: Matrix metallopeptidase 14
NSCLS: Non-small cell lung cancer
P53: Protein 53
PD-L1: Programmed cell death ligand 1
RCC: Renal cell carcinoma
SEC31A: Secretory pathway component 31A
SOCS3: Suppressor cytokine signaling 3
TCF21: Transcription factor 21
TERT: Telomerase reverse transcription
TNRC6A: Trinucleotide repeat containing adaptor 6A
ZEB1: Zinc finger E-Box binding homeobox 1
